# Association of fetal eye movement density with sleeping and developmental problems in 1.5-year-old infants

**DOI:** 10.1038/s41598-022-12330-1

**Published:** 2022-05-17

**Authors:** Kazushige Nakahara, Seiichi Morokuma, Kana Maehara, Hikohiro Okawa, Yasuko Funabiki, Kiyoko Kato

**Affiliations:** 1 Department of Obstetrics and Gynecology, Graduate School of Medical Sciences, Kyushu University, Fukuoka, Japan; 2 Department of Health Sciences, Graduate School of Medical Sciences, Kyushu University, Fukuoka, 812-8582 Japan; 3 Graduate School of Human and Environmental Studies, Kyoto University, Kyoto, Japan; 4 Graduate School of Medicine, Kyoto University, Kyoto, Japan

**Keywords:** Medical research, Predictive markers

## Abstract

Eye movement density (EMD) is an evaluation index of rapid eye movements observed during sleep. This study aimed to investigate the association of fetal EMD with sleeping and developmental problems in infancy. We observed 60 normal singleton pregnancies (gestational age 28–37 weeks) using ultrasonography for 1 h. Fetal eye movements were counted, and EMD was calculated. Participants answered questionnaires regarding their child’s sleep and development 1.5 years after their delivery. The outcomes of an infant’s sleep were night awakening (yes or no), bedtime (before or after 22:00), and nighttime sleep duration (< 9 or ≥ 9 h). An infant’s development was evaluated using the Child Behavior Checklist (CBCL) T-score. We found that decreased fetal EMD was associated with increased night awakening at the age of 1.5 years (odds ratio 0.84, 95% confidence interval 0.69–1.00 per unit decrease in EMD). However, fetal EMD was not associated with bedtime or nighttime sleep duration. In addition, fetal EMD was independently associated with the total problems T-score of the CBCL at the age of 1.5 years in the multivariate model (*p* = 0.047). In conclusion, fetal EMD may be associated with sleep and developmental problems in infants.

## Introduction

Fetal eye movements (EMs) can be observed by ultrasonography from 14 weeks gestation^1^. At 23 weeks gestation, fetal EMs begin to consolidate, and rapid eye movements (REM) may be observed^2^. The cycle of fetal EM and non-EM (NEM) periods emerges from approximately 30 weeks gestation^3,4^. Thus, the development of the sleep cycle is thought to begin during the fetal period^5^.

Eye movement density (EMD) is an evaluation index of REM activity and is reportedly reduced by various factors, such as aging, sleep deprivation, and Parkinson’s disease^6–8^. Children with developmental disorders such as autism spectrum disorder tend to have sleeping problems in early infancy^9–11^. Their REM activity is reportedly lower than that of children with normal development^12,13^. It is possible that these children may already show problems in sleep development during the fetal period. However, there are no reports on the association between fetal EMD and problems in sleep and development after birth.

Because fetal EMD and REM activity cannot be measured directly, we calculated fetal EMD based on EMs observed using ultrasonography. We previously reported that fetal EMD during the EM period increased until 28 weeks gestation and then plateaued until 37 weeks gestation^14^. This study aimed to investigate whether fetal EMD during the plateau period (28–37 weeks gestation) was associated with sleeping and developmental problems in children aged 1.5 years. In addition, we performed stratified analysis to identify which specific period was associated with infant sleep and development. We divided the cases according to the timing of fetal EMD measurement, namely 28–32 weeks gestation and 33–37 weeks gestation (the first and second half of the plateau).

## Results

The baseline characteristics and outcomes of the 60 included cases are shown in Table 1. The mean gestational age at fetal EM data acquisition was 33.4 weeks (28–37 weeks). The average fetal EMD was 9.8/min (standard deviation [SD], 3.2; range 4.2–21.0).

**Table 1:** Baseline characteristics and outcomes of the study population (60 cases).

	Mean	SD	Range
	or		
	n	(%)	
**Maternal characteristics**			
Maternal age at delivery (years)	34.1	5.5	18–46
Parity			
0 (n)	25	(42%)	
≥ 1 (n)	35	(58%)	
**Ultrasonographic measurements**			
Gestational age at examination (weeks)	33.4	3.1	28–37
EM density (n/min)	9.8	3.2	4.2–21.0
**Birth information**			
Gestational age at birth (weeks)	38.5	1.6	34–41
Preterm birth	5	(8%)	
Type of delivery			
Vaginal (n)	40	(67%)	
Cesarean (n)	20	(33%)	
**Birth Weight (g)**	2857	555	1196–3885
Small for gestational age	11	(18%)	
Sex			
Male (n)	32	(53%)	
Female (n)	28	(47%)	
Apgar score at 5 min	9.1	0.4	8–10
pH of the umbilical artery	7.29	0.06	7.12–7.38

SD, standard deviations; EM, eye movement; CBCL, Child Behavior Checklist.

*The items using for analysis and outcome were written in bold font.

### Association between fetal EMD and infant sleep problems

The numbers and proportions of cases with each sleep outcome are as follows: 36 cases (60%) with night awakening, 11 cases (20%) with a bedtime after 22:00, and 22 cases (37%) with nighttime sleep duration of ≤ 9 h (Table 2).

**Table 2:** Association between fetal EMD and infant sleep outcomes.

	No. of answers	No. of outcome		Univariate model			Multivariate model*		
			%	OR	95% CI	*p* value	aOR	95% CI	*p* value
**Night awakening**									
All population	60	36	60	0.84	0.69–1.00	0.046	0.84	0.69–1.00	0.049
Examined between 28 and 32 GWs	23	15	65	0.92	0.62–1.35	0.668	0.97	0.63–1.49	0.878
Examined between 33 and 37 GWs	37	21	57	0.82	0.64–1.00	0.077	0.80	0.61–0.99	0.044
**Bedtime after 22:00**									
All population	55	11	20	1.01	0.80–1.27	0.932	1.00	0.76–1.29	0.975
Examined between 28 and 32 GWs	22	6	27	0.90	0.57–1.37	0.629	0.80	0.38–1.39	0.475
Examined between 33 and 37 GWs	33	5	15	1.15	0.84–1.58	0.369	1.24	0.84–2.00	0.305
**Sleep for ≤ 9 h during the night (20:00–8:00)**									
All population	58	22	37	0.95	0.79–1.12	0.520	0.96	0.80–1.14	0.621
Examined between 28 and 32 GWs	23	11	48	0.96	0.66–1.39	0.844	1.14	0.73–1.88	0.567
Examined between 33 and 37 GWs	35	11	31	0.98	0.78–1.20	0.840	0.99	0.79–1.22	0.920

EMD, eye movement density; OR, odds ratio; aOR, adjusted odds ratio; CI, confidence interval; GWs, gestational weeks.

*Adjusted for maternal age at delivery, parity, and child's birth weight.

**All ORs or aORs are per 1/min increasing fetal EMD.

Fetal EMD was significantly associated with night awakening at the age of 1.5 years (odds ratio [OR] 0.84, 95% confidence interval [CI] 0.69–1.00, *p* = 0.046). Even after adjusting for maternal age, children’s birth weight, and parity (0 or ≥ 1), the association did not change (aOR 0.84, 95% CI 0.69–1.00, *p* = 0.049). On the other hand, fetal EMD was not associated with the other two sleep outcomes, a bedtime after 22:00 and a short nighttime sleep duration.

The stratified analysis showed a significant association between fetal EMD measured from 33 to 37 gestational weeks and infant night waking (OR 0.80, 95% CI 0.61–0.99, *p* = 0.044 in the multivariate model). Conversely, fetal EMD measured from 28 to 32 gestational weeks was not associated with infant night waking.

A sensitivity analysis wherein the “birth weight” covariate was replaced with “gestational weeks at birth” showed no change in the above significant association (Supplementary Table S1).

### Association of fetal EMD and developmental problems

We used the Japanese version of the Child Behavior Check List/1½–5 (CBCL) to evaluate the infants’ developmental problems when they turned 1.5 years old^15^.

The logistic regression model did not show any significant association between fetal EMD and whether the CBCL T-scores for total, internalizing, and externalizing problems were above the cutoff points. The results were similar in both the univariate and multivariate models (Table 3). The results of the stratified analysis by the timing of measurement of fetal EMD were also the same.

**Table 3:** Association between fetal EMD and infant CBCL abnormality.

	No. of answers	No. of outcome		Univariate model			Multivariate model*		
			%	OR	95% CI	*p* value	aOR	95% CI	*p* value
**Total score**									
All population	60	18	30	0.98	0.81–1.16	0.829	0.98	0.81–1.17	0.828
Examined between 28 and 32 GWs	23	8	35	1.25	0.85–1.93	0.278	1.42	0.88–2.58	0.183
Examined between 33 and 37 GWs	37	10	27	0.93	0.72–1.15	0.520	0.92	0.71–1.15	0.480
**Internalizing score**									
All population	60	4	7	0.82	0.51–1.16	0.337	0.79	0.45–1.16	0.316
Examined between 28 and 32 GWs	23	4	17	0.94	0.55–1.53	0.796	0.89	0.49–1.50	0.680
Examined between 33 and 37 GWs	37	0	0	n.a			n.a		
**Externalizing score**									
All population	60	16	27	0.96	0.78–1.15	0.649	0.94	0.76–1.14	0.555
Examined between 28 and 32 GWs	23	4	17	1.12	0.68–1.88	0.657	1.09	0.61–1.99	0.767
Examined between 33 and 37 GWs	37	12	32	0.88	0.68–1.09	0.270	0.87	0.66–1.09	0.257

EMD, eye movement density; OR, odds ratio; aOR, adjusted odds ratio; CI, confidence interval; GWs, gestational weeks.

*Adjusted for maternal age at delivery, parity, and child's birth weight.

**All ORs or aORs are per 1/min increasing fetal EMD.

Linear regression univariate models showed that CBCL T-scores tended to increase as fetal EMD decreased. This was observed in T-scores for total, internalizing, and externalizing problems. However, the differences were not statistically significant (Table 4). After adjusting for maternal age, children’s birth weight, and parity (0 or ≥ 1), fetal EMD was significantly associated with the total problems CBCL T-score (β = − 0.69, 95% CI − 1.36 to − 0.01, *p* = 0.047). The internalizing and externalizing problem T-scores were not associated with fetal EMD before and after adjustment.

**Table 4:** Association of fetal EMD and CBCL T-scores.

	n	Average	Range	Univariate model			Multivariate model*		
				β	95% CI	*p* value	β	95% CI	*p* value
**Total score**									
All population	60	53.9	30–66	− 0.64	− 1.32 to 0.03	0.062	− 0.69	− 1.36 to − 0.01	0.047
Examined between 28 and 32 GWs	23	55.5	38–66	0.32	− 1.08 to 1.73	0.635	0.17	− 1.39 to 1.73	0.825
Examined between 33 and 37 GWs	37	52.9	30–66	− 0.82	− 1.68 to 0.04	0.062	− 0.82	− 1.69 to 0.05	0.064
**Internalizing score**									
All population	60	49.5	38–63	− 0.58	− 1.21 to 0.05	0.071	− 0.60	− 1.24 to 0.05	0.068
Examined between 28 and 32 GWs	23	52.0	38–63	0.13	− 0.35 to 0.60	0.578	0.11	− 0.42 to 0.65	0.654
Examined between 33 and 37 GWs	37	47.9	38–59	− 0.69	− 1.43 to 0.05	0.067	− 0.70	− 1.47 to 0.07	0.072
**Externalizing score**									
All population	60	54.6	36–70	− 0.60	− 1.26 to 0.07	0.077	− 0.64	− 1.30 to 0.01	0.052
Examined between 28 and 32 GWs	23	54.7	43–65	0.20	− 1.03 to 1.43	0.742	0.06	− 1.23 to 1.35	0.925
Examined between 33 and 37 GWs	37	54.5	36–70	− 0.87	− 1.76 to 0.01	0.053	− 0.86	− 1.74 to 0.01	0.054

EMD, eye movement density; β, partial regression coefficient; CI, confidence interval; GWs, gestational weeks.

*Adjusted for maternal age at delivery, parity, and child's birth weight.

The stratified analysis did not show any significant association between CBCL T-scores and a specific period of gestation. However, the results of the second group (33–37 gestational weeks) more closely reflected the overall results compared to the first group (28–32 gestational weeks).

The above significant association persisted even in sensitivity analyses wherein the “birth weight” covariate was replaced with “gestational weeks at birth” (Supplementary Tables S2 and S2).

## Discussion

This study prospectively investigated the association between fetal EMD measured at 28–37 gestational weeks and sleeping and developmental problems in 1.5-year-old infants. This study showed that lower fetal EMD during the fetal EM period was associated with a higher risk of night awakening. Fetal EMD was not associated with whether the CBCL T-scores were above the cutoff points, but lower fetal EMD was associated with higher T-score for total problems in CBCL.

REM sleep develops during the fetal period and is observed from about 30 weeks of gestation^2–5^. REM sleep occupies a large proportion of sleep in full-term neonates, but this gradually decreases with REM activity^5,16^. The present study showed the association between low REM activity during the late fetal period and night awakening at the age of 1.5 years.

Studies with similar results have been reported in premature infants. Decreased EMs and EMD during REM sleep have been reported in premature infants grown to term compared to full-term infants^17^. Premature infants were also reported to have more nocturnal awakenings than full-term infants after the neonatal period^18,19^. One hypothesis that may explain these findings is that early exposure to the extrauterine environment disturbs normal sleep development in premature infants. This is supported by reports that the postnatal environment affects the brain function and structure of premature infants in the neonatal intensive care unit^20,21^. However, altered functional connectivity in the fetal brain was also observed before birth in premature infants^22^. Thus, the intrauterine environment may also have a significant influence on fetal development. Moreover, this influence may continue to affect postnatal neural development. The results of this study suggest that the difference in REM sleep development during the fetal period may also affect postnatal sleep development, even in full-term infants.

In the present study, REM activity during the fetal period was associated with only night awakenings among sleep outcomes in children at 1.5 years of age. A study performed in mice supported this association by describing a common origin of cells that regulate REM/non-REM sleep and wakefulness^23^. This suggests that the factor that affects REM sleep may also affect sleep-awakening.

There have been no reported studies on the association of fetal EM activity with developmental problems after birth. However, the association between REM activity and developmental outcomes has been reported in premature infants at 6 months of age^24^. REM sleep plays an important role in neurological development through synaptogenesis and pruning^25,26^. Therefore, low REM activity during the fetal period may affect development after birth.

CBCL is a developmental screening test that is reportedly associated with the diagnosis of autism; studies show that both sensitivity and specificity of CBCL for diagnosing autism spectrum disorder are approximately 60–80%^27–30^. Children with autism spectrum disorder tend to show REM sleep abnormalities, including a decreased quantity of REM sleep and a lower number of REMs during the REM period^31–34^. Thus, low REM activity during the fetal period may be a prognostic factor and may help identify children who are at high risk for developmental disorders after birth. It may also facilitate early intervention, which is crucial in improving neurodevelopmental outcomes in autism spectrum disorder^35^. In this study, we used sleep and developmental problems in 1.5-year-old infants as the outcome because these problems may also be a precursor to developmental disorders in the future. However, further studies are necessary to investigate whether fetal EMD will be associated with future developmental disorders such as autism.

The associations between fetal EMD and infant outcomes in the present study were insufficient to set the cutoff points for predicting infant outcomes. Fetal EMD was associated with CBCL T-scores, but not with whether these were above the cutoff points. Therefore, the relationship between fetal EMD and infant development observed in this study was very weak, and it could not be concluded whether fetal EMD was an appropriate index for predicting postnatal development problems. In our previous study, fetal EMD did not significantly change within 28–37 gestational weeks^14^. Hence, fetal EMD was measured within this period. However, in this study, fetal EMD measured at 33–37 gestational weeks was observed to be more closely associated with infant outcomes than fetal EMD measured at 28–32 gestational weeks.

The duration of the fetal EM and NEM period increases starting at around 30 weeks of gestation^4^. The same phenomenon has been reported in preterm infants with the same postconceptional age^5^. Since the development of fetal sleep continues beyond the 30th week of pregnancy, it is considered reasonable that EMD in the late fetal period is more closely associated with postnatal sleep and development. In our study, the developmental outcomes were determined from results of a screening questionnaire performed in the postnatal period. Although we could not identify a concrete relationship between fetal EMD and postnatal developmental outcomes, our findings highlight the need to further investigate how fetal EMD may be associated with the clinical diagnosis of developmental disorders. Future studies should explore whether including larger cohorts, measuring fetal EMD only in the late fetal period, and assessing outcomes (developmental disorders) at later stages in life (in older infants) impact this association. Additionally, it may be useful to combine fetal EMD with other clinically known high-risk factors such as parental age.

This study has several limitations. It was conducted in a single institution and had a small sample size. In addition, information about the outcomes in infancy was not available for about half of the original cohort, mainly because of no response to questionnaires. Information about each patient’s family socio-economic status and environment after birth, such as nursery school attendance or the mother’s job, was also not considered. Therefore, we could not fully adjust for factors that may affect infant sleep and development. Also, outcome information for the 1.5-year-old children was acquired from questionnaires completed by their mothers, making results relatively subjective. Conversely, a strong point of this study is that it is the first to describe an association between fetal EMD with sleep and development during infancy.

In conclusion, differences in fetal REM activity in the third trimester may be associated with sleeping and developmental problems in early infancy.

## Methods

### Ethics

This study was conducted in accordance with the Declaration of Helsinki and approved by the Ethics Committee of Kyushu University Hospital (No. 27-51). A written informed consent was obtained from all participants.

### Study participants

**Figure 1:**
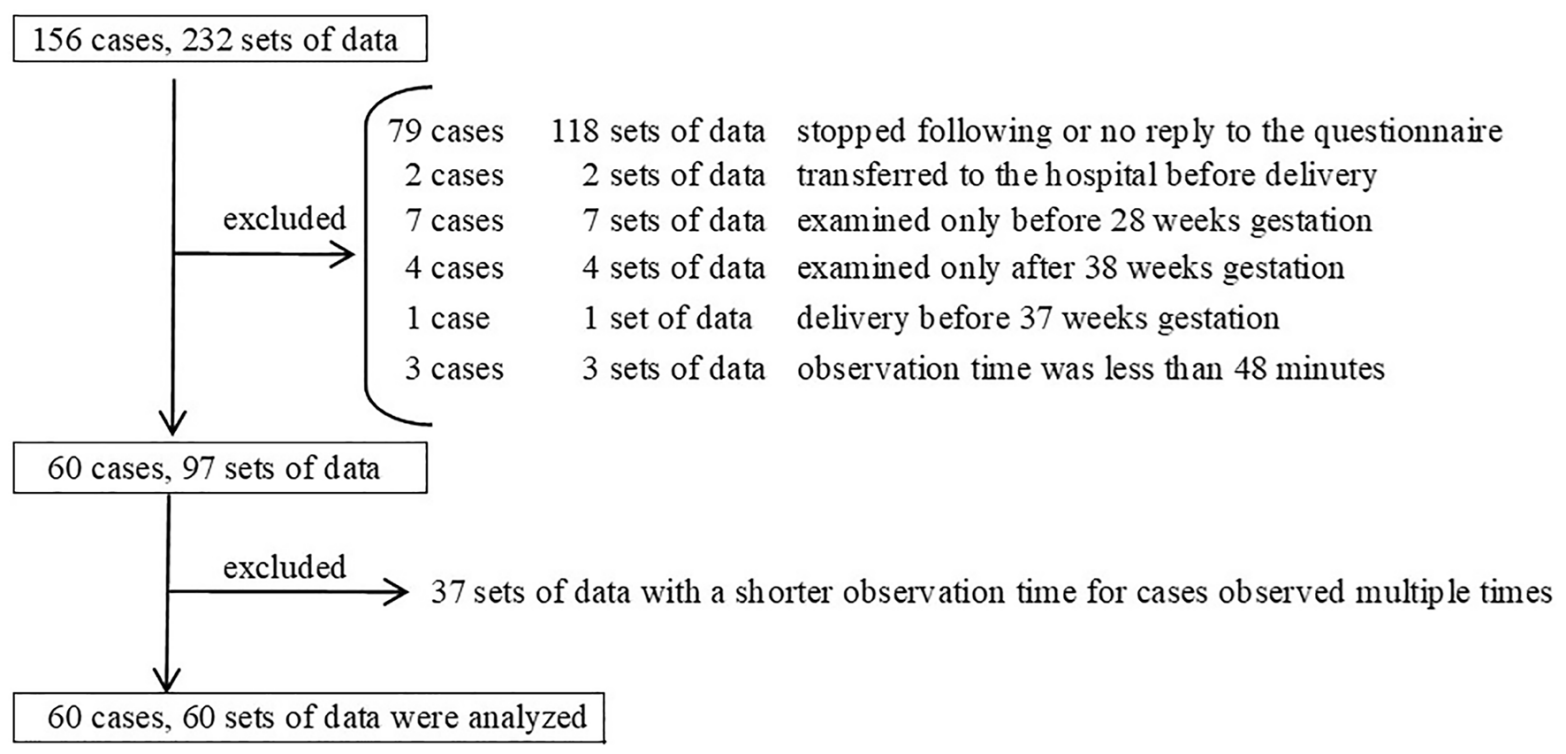
Population flowchart.

To investigate fetal EMs, we conducted 1-h ultrasonography examinations 232 times in 156 cases between 2013 and 2019^14^. We sent questionnaires about infant sleep and development to parents 1.5 years after their delivery. Out of the 156 cases, 96 cases were excluded due to the following reasons: unfollowable or no response to questionnaires (n = 79), transferred to other hospital before delivery (n = 2), examined only before 28 weeks gestation (n = 7), examined only after 38 weeks gestation (n = 4), birth before 34 weeks gestation (n = 1), and effective observation time was less than 48 min (n = 3). The remaining 60 cases were included in the analysis (Fig. 1). In cases where observation was conducted multiple times, we selected the data with the longer effective observation time.

### Data acquisition

Patients were placed in a supine position in a quiet room, allowing them to change positions freely. The procedure was performed between 13:00 and 16:00 at least 2 h after food ingestion. Fetal EMs were observed for 60 min at a frame rate of 30 frames/s or higher using transabdominal two-dimensional sonography (APLIO 500 TUS-A500; TOSHIBA, Japan) with a 3.5-MHz convex transducer (PVT375BT Probe). The video data were recorded in an MP4 format digital video file on an SD card.

### Calculation of fetal EMD

The effective observation time was defined as the time when the fetal EMs could be determined. We excluded 3 cases with data whose effective observation times were ≤ 80% (48 min) from the analysis because accurate measurement of EMD could not be ensured. We counted the fetal EMs (Supplementary Video) and created time-series data of each EM using the video recorded. Each minute was examined, and the periods during which EMs occurred were defined as EM periods. EMD was calculated as the total number of EMs divided by the EM period (minutes). These definitions were the same as in our previous paper^14^.

### Outcome 1: Infant sleep problems

Information regarding each 1.5-year-old infant`s sleep habits was acquired from the questionnaire sent to their mothers. The questionnaire was prepared by modifying the Brief Infant Sleep Questionnaire^36^ and included questions about bedtime, total sleep duration during the night, and nocturnal awakening. Children with autism spectrum disorder report a long sleep latency and frequent nocturnal awakenings^11^. They also tend to wake up earlier and take a longer nap than neurotypical children^10^. Based on these studies, we focused on three points. The first point was night awakening, defined as whether infants usually awoke during the night as the outcome. The second point was the infant`s bedtime. A bedtime later than 22:00 was defined as late. The third point was sleep duration during the night (between 20:00 and 7:59). A sleep duration of ≤ 9 h was deemed unusual. The cutoff points of sleep duration during night and bedtime were determined by referring to the quartile points of the study population.

### Outcome 2: Infant’s developmental problems

We used the Japanese version of the Child Behavior Check List/1½–5 (CBCL) to evaluate an infant`s developmental problems when they turned 1.5 years old. The CBCL is widely used to evaluate emotional/behavioral problems of children^37^, and the version used in the present study has been validated^15^. It consists of 100 items on specific behavior problems scored from 0 (not true at all) to 2 (completely true). The scores per item are added to yield a total problems score and two broadband scores (internalizing and externalizing problems). Internalizing problems comprised emotionally reactive, anxious/depressed, somatic complaints, and withdrawn subscale measures. Externalizing problems comprised attention problems and aggressive problems subscale measures. All scores and subscales are a standardized T-score (mean = 50, SD = 10), and 60 is set as the cutoff score for each domain^15^. The outcomes in this study were defined by whether T-scores were above or below the cutoff points for each CBCL domain. We also used the T-scores as outcomes.

### Covariates

Information about maternal age at delivery, gestational weeks at birth, children’s birth weight, whether or not the infant was small for gestational age, parity (0 or ≥ 1), and infant sex were collected via medical records. We choose these factors as covariates because these were reported as risk factors for developmental disorders^38,39^. Since the number of analysis subjects was limited, we selected three covariates (maternal age, children’s birth weight, and parity) with large correlation coefficients for infant outcomes for main multivariate analysis.

### Statistical analysis

JMP^®^ 14 (SAS Institute Inc., Cary, NC, USA) was used for all analyses. The statistical significance was set at 0.05. Descriptive statistical analyses were performed using means and SD, or numbers and percentages.

To explore the association of fetal EMD and sleeping outcomes, we used a logistic regression model and estimated the OR for each sleeping outcome and 95% CIs. We then used multivariate logistic regression models adjusted for maternal age at delivery, children’s birth weight, and parity (0 or ≥ 1) to estimate the adjusted OR (aOR) and 95% CI.

In the same way as above, we also examined the association between fetal EMD and whether the CBCL scores in total, internalizing, and externalizing problems were above the cutoff points. In addition, we used linear regression models to analyze the association between fetal EMD and the raw CBCL T-scores in total, internalizing, and externalizing problems. The same covariates were selected in the multivariate models used to analyze the association between fetal EMD and sleep outcomes.

We also performed stratified analysis to investigate the period associated with each infant outcome. We divided the cases into those measured at 28–32 gestational weeks (n = 23) and those at 33–37 gestational weeks (n = 37).

In addition, we performed sensitivity analyses by replacing the “birth weight” covariate with “gestational weeks at birth” in the multivariate models.

## Supplementary Information

Supplementary Video 1.

Supplementary Information.

Supplementary Tables.

## Data Availability

The data that support the findings of this study are available from the corresponding author upon reasonable request.
