# Supplementary material for: Association of fetal eye movement density with sleeping and developmental problems in 1.5-year-old infants
Source: Sci Rep. 2022 May 17;12:8236. doi: 10.1038/s41598-022-12330-1 (PMC9114104; doi:10.1038/s41598-022-12330-1)
Supplement: Supplementary file 3 — Supplementary Tables. [file 41598_2022_12330_MOESM3_ESM.docx]

**Association of fetal eye movement density with sleeping and developmental problems in 1.5-year-old infants**

Kazushige Nakahara, Seiichi Morokuma, Kana Maehara, Hikohiro Okawa, Yasuko Funabiki, Kiyoko Kato

**Supplementary Tables**

Supplementary Table S1. Association between fetal EMD and infant's sleep outcomes (changing covariates)

Supplementary Table S2. Association between fetal EMD and abnormal CBCL results in infants (changing covariates)

Supplementary Table S3. Association of fetal EMD and CBCL T-scores (changing covariates)

**Supplementary video**

A sample video identifying and counting fetal eye movements (EMs).

| Supplementary Table S1. Association between fetal EMD and infant's sleep outcomes (changing covariates) | | | | | | | | | | | | | | | |
| --- | --- | --- | --- | --- | --- | --- | --- | --- | --- | --- | --- | --- | --- | --- | --- |
|  |  | No. of answers | No. of outcome | | Original multivariate model* | | | | |  | Sensitivity analysis** | | | | |
|  |  |  |  | % | aOR | 95% CI | | | *p* value |  | aOR | 95% CI | | | *p* value |
| **Night awakening** | |  |  |  |  |  |  |  |  |  |  |  |  |  |  |
|  | All population | 60 | 36 | 60 | 0.84 | 0.69 | to | 1.00 | 0.049 |  | 0.84 | 0.69 | to | 1.00 | 0.048 |
|  | Examined between 28 and 32 GWs | 23 | 15 | 65 | 0.97 | 0.63 | to | 1.49 | 0.878 |  | 0.94 | 0.62 | to | 1.41 | 0.744 |
|  | Examined between 33 and 37 GWs | 37 | 21 | 57 | 0.80 | 0.61 | to | 0.99 | 0.044 |  | 0.79 | 0.60 | to | 0.98 | 0.035 |
|  |  |  |  |  |  |  |  |  |  |  |  |  |  |  |  |
| **Bedtime after 22:00** | |  |  |  |  |  |  |  |  |  |  |  |  |  |  |
|  | All population | 55 | 11 | 20 | 1.00 | 0.76 | to | 1.29 | 0.975 |  | 1.00 | 0.76 | to | 1.28 | 0.990 |
|  | Examined between 28 and 32 GWs | 22 | 6 | 27 | 0.80 | 0.38 | to | 1.39 | 0.475 |  | 0.75 | 0.37 | to | 1.26 | 0.291 |
|  | Examined between 33 and 37 GWs | 33 | 5 | 15 | 1.24 | 0.84 | to | 2.00 | 0.305 |  | 1.24 | 0.85 | to | 1.93 | 0.271 |
|  |  |  |  |  |  |  |  |  |  |  |  |  |  |  |  |
| **Sleep for ≤9 hours during the night (20:00-8:00)** | | | |  |  |  |  |  |  |  |  |  |  |  |  |
|  | All population | 58 | 22 | 37 | 0.96 | 0.80 | to | 1.14 | 0.621 |  | 0.95 | 0.79 | to | 1.13 | 0.597 |
|  | Examined between 28 and 32 GWs | 23 | 11 | 48 | 1.14 | 0.73 | to | 1.88 | 0.567 |  | 1.12 | 0.71 | to | 1.83 | 0.628 |
|  | Examined between 33 and 37 GWs | 35 | 11 | 31 | 0.99 | 0.79 | to | 1.22 | 0.920 |  | 0.98 | 0.78 | to | 1.20 | 0.853 |
| EMD, eye movement density; aOR, adjusted odds ratio; CI, confidence interval; GWs, gestational weeks | | | | | | | | | | |  |  |  |  |  |
| *Adjusted for maternal age at delivery, parity, and child's birth weight | | | | | |  |  |  |  |  |  |  |  |  |  |
| **Adjusted for maternal age at delivery, parity, and **gestational weeks at birth** | | | | | | |  |  |  |  |  |  |  |  |  |
| ***All ORs or aORs are per 1/min increasing fetal EMD | | | |  |  |  |  |  |  |  |  |  |  |  |  |

| Supplemental Table 2. Association between fetal EMDand abnormal CBCL results in infants (changing covariates) | | | | | | | | | | | | | | | |
| --- | --- | --- | --- | --- | --- | --- | --- | --- | --- | --- | --- | --- | --- | --- | --- |
|  |  |  |  |  |  |  |  |  |  |  |  |  |  |  |  |
|  |  | No. of answers | No. of outcome | | Original multivariate model* | | | | |  | Sensitivity analysis** | | | | |
|  |  |  |  | % | OR | 95% CI | | | *p* value |  | aOR | 95% CI | | | *p* value |
| **Total score** | |  |  |  |  |  |  |  |  |  |  |  |  |  |  |
|  | All population | 60 | 18 | 30 | 0.98 | 0.81 | to | 1.17 | 0.828 |  | 0.98 | 0.81 | to | 1.17 | 0.824 |
|  | Examined between 28 and 32 GWs | 23 | 8 | 35 | 1.42 | 0.88 | to | 2.58 | 0.183 |  | 1.37 | 0.87 | to | 2.35 | 0.175 |
|  | Examined between 33 and 37 GWs | 37 | 10 | 27 | 0.92 | 0.71 | to | 1.15 | 0.480 |  | 0.92 | 0.71 | to | 1.15 | 0.492 |
|  |  |  |  |  |  |  |  |  |  |  |  |  |  |  |  |
| **Internalizing score** | |  |  |  |  |  |  |  |  |  |  |  |  |  |  |
|  | All population | 60 | 4 | 7 | 0.79 | 0.45 | to | 1.16 | 0.316 |  | 0.78 | 0.43 | to | 1.17 | 0.260 |
|  | Examined between 28 and 32 GWs | 23 | 4 | 17 | 0.89 | 0.49 | to | 1.50 | 0.680 |  | 0.90 | 0.49 | to | 1.51 | 0.686 |
|  | Examined between 33 and 37 GWs | 37 | 0 | 0 | n.a | | | | |  | n.a | | | | |
|  |  |  |  |  |  |  |  |  |  |  |  |  |  |  |  |
| **Externalizing score** | |  |  |  |  |  |  |  |  |  |  |  |  |  |  |
|  | All population | 60 | 16 | 27 | 0.94 | 0.76 | to | 1.14 | 0.555 |  | 0.94 | 0.76 | to | 1.14 | 0.536 |
|  | Examined between 28 and 32 GWs | 23 | 4 | 17 | 1.09 | 0.61 | to | 1.99 | 0.767 |  | 1.10 | 0.63 | to | 1.99 | 0.720 |
|  | Examined between 33 and 37 GWs | 37 | 12 | 32 | 0.87 | 0.66 | to | 1.09 | 0.257 |  | 0.87 | 0.66 | to | 1.08 | 0.220 |
| EMD, eye movement density; aOR, adjusted odds ratio; CI, confidence interval; GWs, gestational weeks | | | | | | | | | | |  |  |  |  |  |
| *Adjusted for maternal age at delivery, parity, and child's birth weight | | | | | |  |  |  |  |  |  |  |  |  |  |
| **Adjusted for maternal age at delivery, parity, and **gestational weeks at birth** | | | | | | |  |  |  |  |  |  |  |  |  |
| ***All ORs or aORs are per 1/min increasing fetal EMD | | | |  |  |  |  |  |  |  |  |  |  |  |  |

| Supplementary Table S3. Association of fetal EMD and CBCL T-scores (changing covariates) | | | | | | | | | | | | | | | | | |
| --- | --- | --- | --- | --- | --- | --- | --- | --- | --- | --- | --- | --- | --- | --- | --- | --- | --- |
|  |  | n | average | range | | | Original multivariate model* | | | | |  | Sensitivity analysis** | | | | |
|  |  |  |  |  |  |  | β | 95% CI | | | *p* value |  | β | 95% CI | | | *p* value |
| **Total score** | |  |  |  |  |  |  |  |  |  |  |  |  |  |  |  |  |
|  | All population | 60 | 53.9 | 30 | - | 66 | -0.69 | -1.36 | to | -0.01 | 0.047 |  | -0.69 | -1.37 | to | -0.02 | 0.045 |
|  | Examined between 28 and 32 GWs | 23 | 55.5 | 38 | - | 66 | 0.17 | -1.39 | to | 1.73 | 0.825 |  | 0.18 | -1.38 | to | 1.74 | 0.811 |
|  | Examined between 33 and 37 GWs | 37 | 52.9 | 30 | - | 66 | -0.82 | -1.69 | to | 0.05 | 0.064 |  | -0.83 | -1.70 | to | 0.04 | 0.061 |
|  |  |  |  |  |  |  |  |  |  |  |  |  |  |  |  |  |  |
| **Internalizing score** | |  |  |  |  |  |  |  |  |  |  |  |  |  |  |  |  |
|  | All population | 60 | 49.5 | 38 | - | 63 | -0.60 | -1.24 | to | 0.05 | 0.068 |  | -0.60 | -1.25 | to | 0.04 | 0.065 |
|  | Examined between 28 and 32 GWs | 23 | 52.0 | 38 | - | 63 | 0.11 | -0.42 | to | 0.65 | 0.654 |  | 0.43 | -1.21 | to | 2.07 | 0.593 |
|  | Examined between 33 and 37 GWs | 37 | 47.9 | 38 | - | 59 | -0.70 | -1.47 | to | 0.07 | 0.072 |  | -0.70 | -1.45 | to | 0.05 | 0.064 |
|  |  |  |  |  |  |  |  |  |  |  |  |  |  |  |  |  |  |
| **Externalizing score** | |  |  |  |  |  |  |  |  |  |  |  |  |  |  |  |  |
|  | All population | 60 | 54.6 | 36 | - | 70 | -0.64 | -1.30 | to | 0.01 | 0.052 |  | -0.65 | -1.30 | to | 0.00 | 0.050 |
|  | Examined between 28 and 32 GWs | 23 | 54.7 | 43 | - | 65 | 0.06 | -1.23 | to | 1.35 | 0.925 |  | 0.11 | -1.21 | to | 1.43 | 0.863 |
|  | Examined between 33 and 37 GWs | 37 | 54.5 | 36 | - | 70 | -0.86 | -1.74 | to | 0.01 | 0.054 |  | -0.88 | -1.75 | to | 0.00 | 0.049 |
| EMD, eye movement density; β, partial regression coefficient; CI, confidence interval; GWs, gestational weeks | | | | | | | | | | | | | |  |  |  |  |
| *Adjusted for maternal age at delivery, parity, and child’s birth weight | | | | | | | | |  |  |  |  |  |  |  |  |  |
| **Adjusted for maternal age at delivery, parity, and **gestational weeks at birth** | | | | | | | | | | |  |  |  |  |  |  |  |
